# Use of the interRAI CHESS Scale to Predict Mortality among Persons with Neurological Conditions in Three Care Settings

**DOI:** 10.1371/journal.pone.0099066

**Published:** 2014-06-10

**Authors:** John P. Hirdes, Jeffrey W. Poss, Lori Mitchell, Lawrence Korngut, George Heckman

**Affiliations:** 1 School of Public Health and Health Systems, University of Waterloo, Waterloo, ON, Canada; 2 Winnipeg Regional Health Authority, Winnipeg, MB, Canada; 3 Department of Clinical Neurosciences, University of Calgary, Calgary, AB, Canada; Marienhospital Herne - University of Bochum, Germany

## Abstract

**Background:**

Persons with certain neurological conditions have higher mortality rates than the population without neurological conditions, but the risk factors for increased mortality *within* diagnostic groups are less well understood. The interRAI CHESS scale has been shown to be a strong predictor of mortality in the overall population of persons receiving health care in community and institutional settings. This study examines the performance of CHESS as a predictor of mortality among persons with 11 different neurological conditions.

**Methods:**

Survival analyses were done with interRAI assessments linked to mortality data among persons in home care (n = 359,940), complex continuing care hospitals/units (n = 88,721), and nursing homes (n = 185,309) in seven Canadian provinces/territories.

**Results:**

CHESS was a significant predictor of mortality in all 3 care settings for the 11 neurological diagnostic groups considered after adjusting for age and sex. The distribution of CHESS scores varied between diagnostic groups and within diagnostic groups in different care settings.

**Conclusions:**

CHESS is a valid predictor of mortality in neurological populations in community and institutional care. It may prove useful for several clinical, administrative, policy-development, evaluation and research purposes. Because it is routinely gathered as part of normal clinical practice in jurisdictions (like Canada) that have implemented interRAI assessment instruments, CHESS can be derived without additional need for data collection.

## Introduction

The World Health Organization estimates that neurological conditions account for about 12% of global deaths and about 14% of years of healthy life lost to death [1]. For many developed nations, neurological deaths have gained prominence in relation to total mortality in the last three decades [2]. Certain neurological conditions (e.g., multiple sclerosis, stroke, TIA, parkinsonism) are associated with higher risks of mortality rates compared with persons without those conditions [3], [4]; however, the risk factors for increased mortality *within* diagnostic groups are less well understood. Algorithms to predict mortality have been developed specifically for persons with ALS [5], [6], Parkinsons disease [7] and Traumatic Brain Injury [8] using a variety of functional, clinical, and laboratory based indicators. Although some disease-specific methods appear to perform well at predicting survival times, these algorithms are often not applicable across neurological conditions or to non-neurological populations. In addition, the indicators employed in these algorithms are often not readily available in existing medical records.

The Changes in Health, End-stage disease, and Signs and Symptoms (CHESS) scale has been shown to predict mortality, health service use, and caregiver distress in the overall populations of persons receiving care in home care, post-acute, nursing home and palliative care settings [9]–[14]. CHESS is a summary measure based on a count of decline in Activities of Daily Living (ADL); decline in cognition; symptoms such as weight loss, shortness of breath, and edema; and clinician ratings of a prognosis of less than six months. Although counts of deficits [15]–[17] can be useful indicators of frailty in older populations, CHESS has been shown to be a stronger predictor of time to adverse outcomes in home care clients than the Frailty Index [13]. CHESS scores are standardized algorithms obtained from items embedded in the interRAI assessment instruments, which have been adopted across the continuum of care in several countries including Canada [18]–[19]. As a result, persons of different ages receiving care in different service settings can be compared directly with equivalent measures.

While CHESS has been shown to be a good predictor of a variety of adverse outcomes in the overall population of persons receiving non-acute care, it is not clear whether it would function equally well among diagnostic subgroups. Previous research showed that CHESS is a more effective predictor of mortality in nursing home residents with heart failure compared with the New York Heart Association (NYHA) classification [20], but it has not been evaluated specifically for persons with neurological conditions. Given that these data are readily available in countries where interRAI instruments have been adopted as standardized assessments [19], [21], it would be useful to know how the scale performs in specialized subpopulations who would be assessed with the interRAI instrument as part of routine clinical practice. This research was undertaken as part of the National Population Health Study of Neurological Conditions (NPHSNC), which aimed to examine the scope, impact, risk factors and health service use related to neurological conditions in Canada [22].

## Materials and Methods

### Ethics

Ethics clearance for this research was obtained through the Office of Research Ethics at the University of Waterloo.

### Sample

The samples considered in the present analyses include persons in three care settings with 10 neurological conditions identified as priorities for the NPHSNC (i.e., Alzheimer's and related dementia, Parkinson's disease, Traumatic Brain Injury, Epilepsy, Multiple Sclerosis, Cerebral Palsy, Amyotrophic Lateral Sclerosis, Spinal Cord Injury, Muscular Dystrophy, Huntington's Disease), stroke, and other non-neurological conditions. Although stroke was not identified as a priority condition by the funders of NPHSNC, it was retained for the present analyses given its importance as a neurological condition. For some analyses, the 10 priority conditions and stroke are combined as a single neurological conditions group; however, the conditions are also examined separately because it is unlikely that all neurological conditions have the same relationship to mortality.

The samples examined in the present study included Canadian long stay home care (HC) clients in Ontario, Winnipeg Regional Health Authority, and the Yukon Territory (n = 359,940). This included 140,765 persons with one or more of the abovementioned 11 neurological conditions among those home care clients. In addition, data were available for 88,721 Ontario and Winnipeg Regional Health Authority complex continuing care hospital (CCC) patients and 185,309 long term care home (LTC) residents in Ontario, Winnipeg Regional Health Authority, Saskatchewan, Yukon Territory, British Columbia, Nova Scotia, and Newfoundland (50,277 and 146,165 had one or more of the neurological conditions of interest in those settings, respectively).

All persons assessed as part of normal clinical practice with either the Resident Assessment Instrument (RAI 2.0) in CCC or LTC between 2003–2010 or the RAI-Home Care (RAI-HC) in HC settings between 2007-2011 were eligible for inclusion in the study sample. For home care where reassessments are normally conducted between six and 12 months, the last assessment was used. For those in CCC and LTC where 3 month reassessment is done, the observation closest to July 1 in the last year they were assessed was used to construct an observational cohort.

### Data source

The two primary data sources for the study were the Canadian Institute for Health Information's (CIHI) Home Care Reporting System and Continuing Care Reporting System, which house the RAI 2.0 and RAI-HC data on a national basis in Canada. The eight provinces/territories that have mandated implementation of one or more interRAI instruments submit their data to CIHI for national statistical reporting. CIHI in turned linked the assessment data to the hospital Discharge Abstract Database (DAD), National Ambulatory Care Reporting System (NACRS) in order to permit longitudinal follow-up. Mortality was tracked over a six month period following the baseline assessment using these various information sources, and surviving cases were censored at June 30, 2011.

The data used in the present analyses were provided to the research team by CIHI as part of a pre-existing data sharing agreement between interRAI and CIHI. The data are not publicly available and cannot be transmitted to third parties due to legal terms specified in this license agreement; however, other researchers may apply to CIHI for access to the data through another data sharing mechanism available to the general research community in Canada. The data are hosted on a secure server at the University of Waterloo, which meets CIHI's standards for privacy protection. All data were de-identified by CIHI prior to transmission to the University of Waterloo.

The RAI 2.0 and RAI-HC assessments each comprise over 350 sociodemographic, administrative, clinical and diagnostic items at the individual level. In addition to the 11 neurological conditions of interest here, these assessments gather information related to a broad range of functional and clinical measures including the CHESS scale. Evidence about the reliability and validity of the interRAI family of assessment instruments has been reported elsewhere [23]–[27], including reports on the validity of diagnostic data in these assessments [28]–[29].

### Statistical analysis

The first analytic steps focused on the description of the study samples in each of the three care settings of interest by diagnosis with the aim of understanding differences in the distributions of underlying risk factors for mortality between the diagnostic groups (e.g., age, sex). The diagnostic groups were coded as a series of binary variables that were not mutually exclusive (e.g., persons with traumatic brain injury and epilepsy were coded as having the diagnosis “present” for both binary variables); however, the group “None of these 11 conditions” had none of the 11 neurological conditions of interest present. In addition, the relationships between diagnosis and clinical characteristics like cognitive impairment, physical disability, depression and CHESS scores were also examined.

Proportional hazards models were used to examine mortality within each of the neurological subgroups and among persons with none of the neurological conditions of interest. In addition, the survival rates for all persons in each of the HC, LTC and CCC settings were examined by neurological condition after controlling for CHESS, age, and sex. It was not the aim of the present study to develop definitive, comprehensive models of all risk factors for mortality within each neurological condition, but rather to explore the utility of the CHESS scale for persons with neurological conditions. All diagnoses were included in the multivariate analyses as dummy variables, which would permit persons with multiple neurological diagnoses to be included in the model and would permit estimates of the independent explanatory power of individual diagnoses after adjusting for comorbid conditions.

In addition, logistic regression models using the same independent variables employed in the survival models were used to model 6-month mortality as a binary dependent variable. The c statistics obtained from these models were used to provide information on the explanatory power of the models and their components.

The 6-month follow-up period for mortality was chosen to reflect the recommended interval between assessments in home care. In the other two settings, a shorter reassessment cycle (three months) is used. Therefore, half a year is the longest recommended time period without a follow-up assessment that would provide new information to inform clinical action. In other words, the time frame used in the survival models reflects the period for which assessments are intended to inform clinical responses to the person's needs before being reassessed.

In addition to the hazard ratios provided by the survival models, estimates of the death rate per 1,000 person years were derived to illustrate the magnitude of mortality across settings and between diagnostic groups.

The analyses were done for the three care settings separately, rather than as a pooled sample, because the individual sectors would be particularly interested in the applicability of the CHESS within their own setting. In addition, the mortality rates differ substantially between settings for all cases combined and for individual diagnostic groups.

## Results

Table 1 provides a summary of the demographic and clinical characteristics of the samples of persons with neurological conditions and those with none of the 11 conditions of interest in all three care settings. Substantial differences in the age and sex distributions by diagnosis are evident within settings; however, these differences also occur within diagnostic groups in different care settings. For example persons with cerebral palsy and muscular dystrophy tend to be youngest and those with Alzheimer's and related dementias are the oldest in each care setting. However, for each diagnostic group, those in HC and CCC tend to be younger than in LTC. In addition, persons with multiple sclerosis have the highest percentage of females in all three settings.

**Table 1:** Percentage distribution of selected demographic and clinical characteristics by diagnosis and care setting in five Canadian provinces/territories.

Setting and Diagnosis		Age Group (%)				Selected Characteristics (%)			
	n	<65	65–74	75–84	85+	Female	CPS^1^ 3+	ADL 3+	DRS 3+
***Home care***									
All HC clients	359,940	16.6	14.8	34.4	34.2	62.9	16.1	20.2	16.1
Amyotrophic Lateral Sclerosis	1,264	31.7	28.0	31.2	9.1	44.6	16.4	58.1	23.4
Cerebral Palsy	1,413	77.7	11.1	7.5	3.7	52.0	37.3	61.1	12.8
Alzheimer's & Related Dementias	106,603	3.1	9.8	40.9	46.2	62.3	43.0	33.4	19.2
Epilepsy	9,716	38.2	19.4	26.6	15.9	54.2	31.6	37.2	18.3
Huntington's Disease	304	58.1	20.8	16.2	5.0	59.2	39.1	42.1	19.4
Muscular Dystrophy	4,852	64.9	19.6	11.9	3.7	70.0	7.7	40.9	18.1
Multiple Sclerosis	696	72.7	13.7	10.1	3.5	47.4	6.3	44.8	13.7
Parkinson's Disease	17,915	6.1	17.4	48.1	28.4	46.8	25.9	38.2	19.0
Spinal Cord Injury	794	58.5	16.6	16.0	9.0	31.2	5.7	49.2	14.9
Stroke	88,549	9.1	14.4	38.3	38.2	58.7	22.5	29.5	16.7
Traumatic Brain Injury	13,722	24.0	13.9	31.0	31.2	54.8	25.1	27.9	18.9
None of these 11 conditions	178,369	22.7	16.9	31.1	29.3	65.2	2.9	10.7	14.4
***Complex continuing care hospitals/units***									
All CCC patients	88,721	16.5	17.9	36.4	29.2	55.7	43.1	72.8	22.8
Amyotrophic Lateral Sclerosis	654	31.7	25.7	31.2	11.5	45.4	48.5	91.1	30.4
Cerebral Palsy	531	60.1	14.9	16.0	9.0	43.5	59.7	88.5	21.1
Alzheimer's & Related Dementias	26,336	5.5	11.5	41.1	41.9	53.4	72.8	78.7	28.6
Epilepsy	5,880	38.9	21.0	25.9	14.2	49.1	62.3	81.7	25.7
Huntington's Disease	184	57.6	16.3	16.9	9.2	52.2	83.7	91.9	27.2
Muscular Dystrophy	1,213	61.3	19.9	14.6	4.2	64.2	40.2	87.6	24.6
Multiple Sclerosis	175	73.7	10.9	10.9	4.6	45.7	28.0	84.0	26.0
Parkinson's Disease	4,622	5.7	16.7	50.2	27.4	42.4	58.5	80.0	23.4
Spinal Cord Injury	2,875	49.2	19.4	19.8	11.6	42.8	50.8	94.4	27.4
Stroke	26,874	12.3	17.8	38.8	31.2	52.5	52.9	76.5	21.8
Traumatic Brain Injury	3,974	30.3	17.2	29.0	23.5	45.8	56.2	73.2	22.4
None of these 11 conditions	38,444	18.4	20.5	35.8	25.3	59.3	24.9	67.4	20.3
***Nursing homes***									
All NH residents	185,309	5.4	8.6	30.7	55.3	67.5	59.9	73.6	28.8
Amyotrophic Lateral Sclerosis	652	14.6	22.6	33.3	29.6	55.2	49.7	87.6	30.8
Cerebral Palsy	2,359	24.9	15.4	27.3	32.4	58.5	73.1	88.3	24.4
Alzheimer's & Related Dementias	118,429	2.6	7.4	32.7	57.4	68.3	74.4	77.9	30.5
Epilepsy	11,407	18.6	17.9	33.4	30.2	57.4	65.4	80.3	28.9
Huntington's Disease	469	50.3	16.0	22.8	10.9	60.8	74.2	83.2	23.9
Muscular Dystrophy	3,588	33.1	19.6	24.8	22.6	62.2	48.0	88.8	28.0
Multiple Sclerosis	185	45.4	17.8	20.0	16.8	56.2	30.8	79.5	38.4
Parkinson's Disease	13,748	4.5	12.4	42.0	41.2	53.7	61.7	83.5	27.7
Spinal Cord Injury	1,421	32.2	17.2	28.2	22.3	51.2	45.3	93.9	27.9
Stroke	52,156	4.5	9.7	33.8	52.1	63.5	58.9	78.1	28.9
Traumatic Brain Injury	8,500	16.5	13.0	30.2	40.3	56.9	63.6	74.6	28.3
None of these 11 conditions	39,144	6.2	8.5	25.0	60.3	69.6	33.4	60.4	26.0

1 CPS – Cognitive Performance Scale (range 0–6 with higher scores indicating more impairment); ADL – Activities of Daily Living Hierarchy Scale (range 0–6 with higher scores indicating more impairment); DRS – Depression Rating Scale (range 0–14 with higher scores indicating more frequent and severe depressive symptoms).

There are also substantial differences in cognitive impairment, functional status, depressive symptoms and CHESS scores across settings and between diagnostic groups (see Tables 1 and 2). Compared with persons without any of the 11 conditions of interest, the neurological subgroups have higher rates of moderate or worse cognitive and ADL impairment. Less pronounced differences are evident for depressive symptoms. However, for all three of these clinical issues the rates are lower in HC compared with facility based settings. On the other hand, the percentage with CHESS scores of two or more are lowest in LTC homes and highest in CCC hospitals/units, and these trends are evident within diagnostic groups across settings (Table 2).

**Table 2:** Percentage distribution of the CHESS scale by diagnosis and care setting in five Canadian provinces/territories.

Setting and Diagnosis		CHESS^1^ Score (%)					
		0	1	2	3	4	5
***Home care***							
All HC clients	359,940	27.2	31.3	25.6	12.3	3.4	0.3
Amyotrophic Lateral Sclerosis	1,264	13.3	32.9	31.8	17.6	3.8	0.5
Cerebral Palsy	1,413	51.8	29.4	14.6	3.5	0.7	0.0
Alzheimer's & Related Dementias	106,603	21.9	27.7	31.2	13.9	5.1	0.3
Epilepsy	9,716	29.4	29.9	25.9	11.4	3.1	0.3
Huntington's Disease	304	28.6	34.3	28.6	6.4	2.1	0.0
Muscular Dystrophy	4,852	34.3	36.4	21.1	6.9	1.2	0.1
Multiple Sclerosis	696	31.8	39.2	20.0	8.7	0.3	0.0
Parkinson's Disease	17,915	22.1	32.2	29.4	12.4	3.8	0.2
Spinal Cord Injury	794	42.6	35.6	14.9	6.1	0.8	0.0
Stroke	88,549	25.3	30.0	27.3	13.1	4.0	0.3
Traumatic Brain Injury	13,722	28.5	29.8	25.9	12.1	3.5	0.2
None of these 11 conditions	178,369	25.0	29.8	28.1	12.8	4.1	0.3
***Complex continuing care hospitals/units***							
All CCC patients	88,721	15.4	20.7	22.2	17.9	14.6	9.3
Amyotrophic Lateral Sclerosis	654	19.0	23.7	26.0	15.9	10.9	4.6
Cerebral Palsy	531	34.1	28.1	22.0	8.7	4.9	2.3
Alzheimer's & Related Dementias	26,336	15.6	19.4	23.9	19.4	15.1	6.6
Epilepsy	5,880	19.1	21.1	21.1	16.1	13.1	9.6
Huntington's Disease	184	29.4	26.6	20.1	10.3	8.7	4.9
Muscular Dystrophy	1,213	31.4	30.1	18.4	10.1	6.9	3.1
Multiple Sclerosis	175	26.3	27.4	30.9	10.3	4.0	1.1
Parkinson's Disease	4,622	17.7	22.5	24.2	18.5	12.7	4.5
Spinal Cord Injury	2,875	27.4	24.7	19.4	11.7	9.5	7.3
Stroke	26,874	18.6	22.5	24.1	16.6	12.4	5.7
Traumatic Brain Injury	3,974	24.3	23.1	23.6	15.4	9.9	3.7
None of these 11 conditions	38,444	17.9	21.6	23.4	17.3	13.3	6.6
***Nursing homes***							
All NH residents	185,309	43.0	29.9	16.7	6.4	3.2	0.8
Amyotrophic Lateral Sclerosis	652	44.2	28.2	18.3	5.7	3.4	0.3
Cerebral Palsy	2,359	45.4	27.0	17.5	6.3	3.1	0.7
Alzheimer's & Related Dementias	118,429	43.5	30.1	16.3	6.3	3.0	0.8
Epilepsy	11,407	46.4	30.0	14.8	5.4	2.6	0.8
Huntington's Disease	469	55.7	25.4	12.2	4.5	1.9	0.4
Muscular Dystrophy	3,588	43.8	29.6	16.7	6.4	3.2	0.3
Multiple Sclerosis	185	49.7	27.6	16.2	3.8	2.2	0.5
Parkinson's Disease	13,748	45.1	30.3	15.8	5.5	2.6	0.8
Spinal Cord Injury	1,421	46.2	33.0	14.6	4.2	1.6	0.4
Stroke	52,156	45.3	30.0	15.4	5.5	2.9	0.9
Traumatic Brain Injury	8,500	47.5	29.1	14.5	5.5	2.7	0.7
None of these 11 conditions	39,144	44.2	30.0	16.1	6.1	2.9	0.8

1 CHESS – Changes in health, signs and symptoms, and end-stage disease (range 0-5 with higher scores indicating more health instability).

These clinical and demographic differences indicate that there is substantial heterogeneity within and between persons with different types of neurological conditions across the continuum of care that would not be taken into account if one considered diagnosis alone. Table 3 provides the age and sex-adjusted hazard ratios and 95% confidence limits for the CHESS scale for each neurological condition in the three care settings. For almost all conditions in HC, LTC, and CCC there were consistent increments in the hazard ratios for six month mortality by CHESS score within the diagnostic group. In a few cases, small cell sizes resulted in confidence intervals overlapping with 1.00. Only Huntington's disease failed to show a strong association between CHESS scores and survival in HC and CCC; however, higher CHESS scores were strongly associated with higher hazard ratios for mortality among residents with Huntington's in LTC.

**Table 3:** Age- and sex-adjusted hazard ratios for mortality by CHESS score, setting and diagnosis in five Canadian provinces/territories.

*Setting and Diagnosis*		Age-Sex Adjusted Hazard Ratio (95% CI) by CHESS Score (Ref = 0)				
	n	1	2	3	4	5
***Home care***						
All HC clients	359,940	1.51	2.22	3.87	6.57	18.98
		(1.46–1.55)	(2.15–2.29)	(3.75–4.00)	(6.31–6.83)	(17.57–20.51)
Amyotrophic Lateral Sclerosis	1,264	2.05	2.86	3.53	6.69	13.38
		(1.23–3.39)	(1.74–4.69)	(2.10–5.94)	(3.61–12.43)	(4.49–39.88)
Cerebral Palsy	1,413	1.85	2.13	4.02	19.11	——1
		(0.99–3.47)	(1.01–4.48)	(1.37–11.86)	(5.42–67.40)	
Alzheimer's & Related Dementias	106,603	1.36	1.61	2.65	4.24	13.96
		(1.28–1.44)	(1.52–1.70)	(2.50–2.82)	(3.95–4.55)	(11.82–16.47)
Epilepsy	9,716	1.28	1.83	2.85	4.37	28.63
		(1.03–1.59)	(1.49–2.25)	(2.27–3.57)	(3.24–5.89)	(17.70–46.30)
Huntington's Disease	304	1.14	2.16	3.87	3.49	——
		(0.25–5.18)	(0.53–8.72)	(0.64–23.28)	(0.32–38.33)	
Muscular Dystrophy	4,852	0.94	1.42	3.84	5.84	14.88
		(0.69–1.28)	(1.04–1.96)	(2.74–5.39)	(3.35–10.18)	(5.34–41.49)
Multiple Sclerosis	696	1.39	2.40	2.61	——	——
		(0.76–2.53)	(1.27–4.54)	(1.18–5.80)		
Parkinson's Disease	17,915	1.17	1.51	2.32	3.54	12.37
		(1.03–1.34)	(1.32–1.72)	(2.01–2.69)	(2.94–4.27)	(7.70–19.88)
Spinal Cord Injury	794	1.36	1.10	3.74	5.03	——
		(0.69–2.70)	(0.45–2.73)	(1.61–8.70)	(1.09–23.23)	
Stroke	88,549	1.33	1.71	2.62	4.00	13.54(
		(1.26–1.41)	(1.62–1.80)	(2.47–2.78)	(3.71–4.32)	11.48–15.97)
Traumatic Brain Injury	13,722	1.25	1.60	2.32	3.73	16.65
		(1.08–1.45)	(1.38–1.85)	(1.98–2.73)	(3.04–4.58)	(9.89–28.02)
None of these 11 conditions	219,175	1.60	2.69	4.76	9.11	20.94
		(1.54–1.66)	(2.59–2.80)	(4.57–4.95)	(8.67–9.58)	(19.11–22.94)
***Complex Continuing Care Hospitals/Units***						
All CCC patients	88,721	1.58	2.52	4.71	9.14	21.07
		(1.49–1.67)	(2.39–2.66)	(4.47–4.96)	(8.68–9.63)	(19.98–22.21)
Amyotrophic Lateral Sclerosis	654	1.18	1.87	2.52	5.09	8.73
		(0.80–1.74)	(1.30–2.68)	(1.73–3.67)	(3.39–7.63)	(5.33–14.29)
Cerebral Palsy	531	1.08	1.61	3.65	6.26	18.33
		(0.64–1.82)	(0.97–2.66)	(2.01–6.64)	(3.14–12.47)	(8.67–38.76)
Alzheimer's & Related Dementias	26,336	1.57	2.03	3.25	5.74	16.64
		(1.43–1.73)	(1.85–2.21)	(2.98–3.54)	(5.26–6.26)	(15.19–18.22)
Epilepsy	5,880	1.80	2.37	4.47	8.86	17.09
		(1.50–2.16)	(1.99–2.84)	(3.76–5.32)	(7.47–10.51)	(14.39–20.29)
Huntington's Disease	184	1.33	1.66	0.87	1.30	32.46
		(0.72–2.46)	(0.84–3.28)	(0.34–2.17)	(0.48–3.51)	(11.88–88.72)
Muscular Dystrophy	1,213	1.20	2.08	2.98	6.92	20.10
		(0.88–1.65)	(1.49–2.90)	(2.07–4.29)	(4.89–9.80)	(13.11–30.81)
Multiple Sclerosis	175	1.53	5.58	6.26	9.05	8.40
		(0.40–5.87)	(1.82–17.11)	(1.77–22.11)	(1.95–42.06)	(0.90–78.18)
Parkinson's Disease	4,622	1.24	1.98	2.98	5.46	21.43
		(0.99–1.55)	(1.62–2.43)	(2.42–3.65)	(4.44–6.70)	(17.08–26.88)
Spinal Cord Injury	2875	1.56	2.44	4.75	10.02	20.79
		(1.27–1.91)	(2.00–2.99)	(3.86–5.85)	(8.12–12.35)	(16.67–25.94)
Stroke	26,874	1.46	2.08	3.63	6.88	20.48
		(1.33–1.61)	(1.90–2.28)	(3.32–3.97)	(6.29–7.51)	(18.67–22.48)
Traumatic Brain Injury	3,974	1.60	2.05	3.92	6.54	23.19
		(1.25–2.04)	(1.62–2.60)	(3.10–4.95)	(5.15–8.30)	(17.90–30.04)
None of these 11 conditions	38,444	1.57	2.99	5.87	11.42	21.74
		(1.41–1.75)	(2.71–3.30)	(5.32–6.46)	(10.38–12.56)	(19.75–23.94)
***Nursing Homes***						
All NH residents	185,309	1.57	2.43	3.92	6.69	22.76
		(1.53–1.60)	(2.37–2.49)	(3.81–4.04)	(6.46–6.93)	(21.53–24.06)
Amyotrophic Lateral Sclerosis	652	1.33	2.01	2.50	7.81	——
		(0.91–1.93)	(1.37–2.94)	(1.42–4.40)	(4.28–14.24)	
Cerebral Palsy	2,359	1.41	1.82	2.65	3.50	7.96
		(1.19–1.69)	(1.51–2.20)	(2.08–3.38)	(2.59–4.72)	(4.72–13.42)
Alzheimer's & Related Dementias	118,429	1.53	2.29	3.66	6.22	24.08
		(1.49–1.57)	(2.22–2.36)	(3.52–3.80)	(5.94–6.51)	(22.39–25.89)
Epilepsy	11,407	1.65	2.41	3.59	6.42	20.29
		(1.51–1.81)	(2.17–2.67)	(3.14–4.10)	(5.48–7.52)	(16.12–25.54)
Huntington's Disease	469	2.73	3.01	7.13	8.23	37.95
		(1.69–4.40)	(1.67–5.43)	(3.37–15.09)	(3.12–21.67)	(8.44–170.70)
Muscular Dystrophy	3,588	1.50	2.11	3.86	5.34	17.80
		(1.27–1.78)	(1.76–2.52)	(3.12–4.76)	(4.12–6.93)	(9.11–34.78)
Multiple Sclerosis	185	0.98	1.98	11.63	14.70	——
		(0.49–1.97)	(0.89–4.41)	(3.74–36.21)	(2.95–73.18)	
Parkinson's Disease	13,748	1.49	2.41	4.02	6.93	26.16
		(1.37–1.62)	(2.20–2.63)	(3.58–4.50)	(6.01–7.99)	(21.34–32.06)
Spinal Cord Injury	1421	1.44	1.71	5.48	6.16	14.15
		(1.12–1.85)	(1.26–2.31)	(3.82–7.86)	(3.53–10.74)	(6.19–32.37)
Stroke	52,156	1.51	2.34	4.02	7.07	27.74
		(1.45–1.58)	(2.23–2.45)	(3.78–4.27)	(6.59–7.58)	(25.06–30.72)
Traumatic Brain Injury	8,500	1.71	2.60	4.14	7.20	34.96
		(1.52–1.91)	(2.29–2.94)	(3.54–4.83)	(5.97–8.68)	(26.46–46.19)
None of these 11 conditions	39,144	1.62	2.60	4.02	6.77	18.64
		(1.55–1.70)	(2.48–2.73)	(3.79–4.26)	(6.33–7.25)	(16.73–20.75)

1 Cells with double dashes do not have hazard ratios due to small cell sizes.

The magnitude of changes in hazard ratios for each increment in the CHESS scale varied somewhat by diagnostic group. For example, the increase in hazard ratios for persons with CP in LTC homes was significant, but much less pronounced than for other diagnostic groups in those settings. Also, the hazard ratios for CHESS scores of 5 differed by diagnosis, but they were consistently associated with dramatically higher hazard ratios compared with the reference group of CHESS equal to 0. The small cell sizes for some conditions (e.g., Muscular Dystrophy) result in estimates with wide confidence intervals, but the general trend of increased hazard ratios with higher CHESS scores is quite consistent.

**Figure 1:**
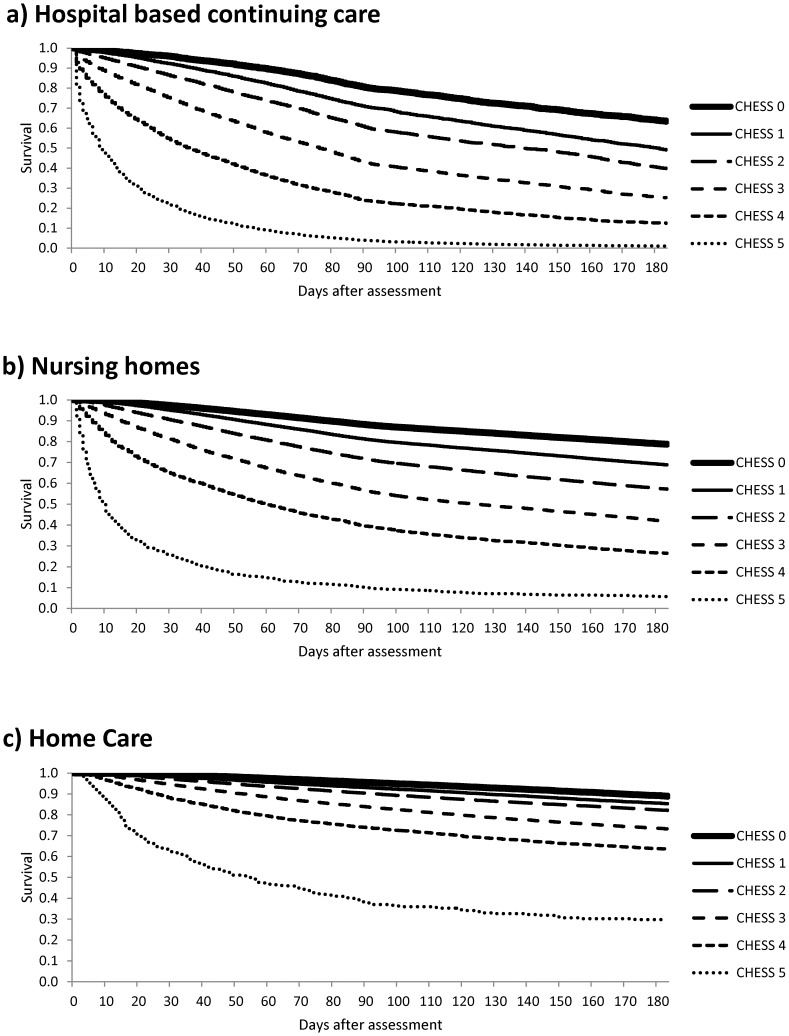
Survival curves for persons with any of 11 neurological conditions by CHESS score in hospital based continuing care (1a), nursing homes (1b), or home care (1c).

Figures 1a to 1c provide the survival curves for persons with any of the 11 neurological conditions by CHESS score across the three care settings. In each case, the CHESS score differentiated the survival experience of persons with neurological conditions. However, the differences in survival by CHESS were largest in facility based settings and survival rates were generally the lowest in CCC hospitals/units.

Table 4 provides the results for multivariate proportional hazards models for six month survival by care setting using diagnosis as a covariate rather than a stratification variable. In HC and LTC homes, age was associated with an increased risk of mortality, but this was not evident in the CCC sample. On the other hand, being female was a protective factor in each setting, but only slightly so in CCC. In all three settings, higher CHESS scores were related to higher hazard ratios for mortality after adjusting for age, sex and diagnosis. However, the various neurological diagnoses were only weakly (and inconsistently) related to mortality after adjusting for age, sex, and CHESS score. Only ALS was consistently associated with an elevated mortality risk, but Alzheimer's and related dementias and Parkinson's were consistently negatively associated with mortality in the adjusted models. The other diagnostic groups were either not significantly different than the reference group or they had inconsistent patterns across care settings. Table 5 provides the c statistics (and 95% confidence limits) obtained from logistic regression models for mortality as a binary outcome within a 6-month period using the same independent variables as the survival models in Table 4. In each care setting, the value of c was modest (<0.70) for the baseline models with the 11 diagnostic variables, age and sex; however, the addition of CHESS substantially improved the performance of these models in all three care settings.

**Table 4:** Unadjusted and adjusted hazard ratios controlling for age, sex and CHESS score by diagnosis and care setting in five Canadian provinces.

Independent Variable	Home Care (n = 359,940)		CCC hospitals/units (n = 88,721)		Nursing homes (n = 185,309)	
	Unadjusted HR	Adjusted HR	Unadjusted HR	Adjusted HR	Unadjusted HR	Adjusted HR
**Age group (ref<65)**						
65–74		1.38 (1.33–1.43)		1.12 (1.07–1.16)		1.51 (1.42–1.62)
75–84		1.48 (1.43–1.53)		1.07 (1.04–1.11)		2.05 (1.93–2.17)
85+		1.76 (1.70–1.82)		1.12 (1.08–1.16)		2.66 (2.51–2.81)
**Female**		0.65 (0.64–0.66)		0.86 (0.85–0.88)		0.74 (0.73–0.76)
**CHESS score (ref = 0)**						
1		1.51 (1.46–1.55)		1.54 (1.45–1.63)		1.57 (1.53–1.60)
2		2.28 (2.21–2.35)		2.46 (2.33–2.59)		2.42 (2.36–2.48)
3		3.93 (3.81–4.06)		4.53 (4.30–4.78)		3.90 (3.79–4.02)
4		6.90 (6.64–7.18)		8.72 (8.27–9.18)		6.70 (6.47–6.94)
5		18.80 (17.40–20.32)		19.32 (18.32–20.38)		22.40 (21.18–23.68)
**Amyotrophic Lateral Sclerosis**	2.14 (1.92–2.39)	1.97 (1.76–2.20)	0.98 (0.88–1.09)	1.19 (1.07–1.33)	1.00 (0.87–1.15)	1.12 (0.97–1.29)
**Cerebral Palsy**	0.35 (0.27–0.45)	0.59 (0.46–0.77)	0.43 (0.37–0.52)	0.78 (0.65–0.92)	1.23 (1.15–1.31)	1.35 (1.26–1.44)
**Alzheimer's & Related Dementias**	0.93 (0.91–0.95)	0.74 (0.72–0.75)	0.78 (0.76–0.80)	0.83 (0.81–0.85)	0.86 (0.85–0.88)	0.84 (0.82–0.85)
**Epilepsy**	0.70 (0.65–0.75)	0.78 (0.72–0.83)	0.81 (0.85–0.92)	1.01 (0.97–1.10)	0.88 (0.85–0.91)	1.05 (1.01–1.09)
**Huntington's Disease**	0.45 (0.27–0.73)	0.63 (0.39–1.04)	0.66 (0.53–0.82)	0.93 (0.75–1.16)	0.68 (0.56–0.83)	1.15 (0.94–1.39)
**Muscular Dystrophy**	1.02 (0.82–1.26)	1.36 (1.09–1.70)	0.35 (0.25–0.48)	0.49 (0.36–0.69)	0.97 (0.74–1.27)	1.33 (1.01–1.75)
**Multiple Sclerosis**	0.46 (0.41–0.51)	0.66 (0.59–0.74)	0.56 (0.50–0.62)	0.87 (0.78–0.96)	0.91 (0.85–0.97)	1.09 (1.02–1.16)
**Parkinson's Disease**	0.93 (0.90–0.97)	0.86 (0.82–0.90)	0.71 (0.67–0.74)	0.85 (0.80–0.89)	0.91 (0.88–0.94)	0.96 (0.93–0.99)
**Spinal Cord Injury**	0.51 (0.39-0.67)	0.68 (0.52–0.89)	0.76 (0.72–0.81)	1.13 (1.07–1.20)	0.92 (0.84–1.02)	1.18 (1.06–1.30)
**Stroke**	1.13 (1.11–1.15)	1.05 (1.03–1.07)	0.64 (0.62–0.65)	0.80 (0.78–0.82)	0.85 (0.83–0.87)	0.89 (0.87–0.91)
**Traumatic Brain Injury**	0.95 (0.91–1.00)	1.00 (0.95–1.46)	0.50 (0.47–0.53)	0.69 (0.64–0.73)	0.80 (0.77–0.84)	0.93 (0.89–0.97)

**Table 5:** C statistics (95% CL) for multiple logistic regression models including age, sex, diagnoses and CHESS score, by care setting in five Canadian provinces/territories.

Model	Home Care	Nursing Homes	Complex Continuing Care Hospitals/Units
Adjusted for age, sex, diagnoses	0.622 (0.619, 0.625)	0.608 (0.605, 0.611)	0.649 (0.645, 0653)
Adjusted for age, sex, diagnoses, CHESS score	0.752 (0.749, 0.755)	0.713 (0.710, 0.716)	0.829 (0.826, 0.832)

Table 6 provides the number of deaths per 1,000 person years by CHESS and care setting in order to describe the magnitude of mortality for selected diagnoses in the study samples. As might be expected, mortality tends to be lowest in home care settings and highest in complex continuing care hospitals/units. When mortality was considered within sectors, the number of deaths per 1,000 person years was reasonably comparable within CHESS levels across the diagnostic groups. The variations evident would be explained, at least in part, by the lack of age and sex adjustments for these rates.

**Table 6:** Deaths per 1,000 person-years by CHESS score, diagnosis and care setting in five Canadian provinces/territories.

Diagnosis	CHESS Score	Home Care	Nursing Homes	Complex Continuing Care Hospitals/Units
		Rate (95% CL)	Rate (95% CL)	Rate (95% CL)
**Alzheimer's and Related Dementia**	0	221 (209–232)	491 (481–501)	937 (848–1,030)
	1	302 (290–315)	762 (745–778)	1,495 (1,383–1,614)
	2	359 (346–372)	1,153 (1,124–1,183)	1,953 (1,829–2,083)
	3	597 (570–624)	1,878 (1,807–1,951)	3,179 (2,983–3,387)
	4	957 (897–1,020)	3,244 (3,069-3,430)	5,751 (5,388–6,141)
	5	3,113 (2,483–3,910)	12,547 (11,054–14,409)	17,478 (16,145–19,007)
	All	371 (363–378)	802 (793–812)	2,673 (2,596–2,752)
**Multiple Sclerosis**	0	135 (104–167)	448 (390–507)	869 (634–1,148)
	1	126 (97–157)	722 (632–817)	1,050 (779–1,375)
	2	201 (152–253)	1,116 (959–1,288)	1,882 (1,332–2,599)
	3	545 (393–715)	2,187 (1,780–2,676)	2,947 (1,968–4,381)
	4	904 (429–1,560)	3,105 (2,308–4,206)	6,703 (4,616–10,238)
	5	2,910 (209–45,737)	10,084 (3,927–47,131)	22,400 (15,269–38,440)
	All	181 (159–203)	774 (722–829)	1,648 (1,431–1,888)
**Parkinson's Disease**	0	248 (219–277)	476 (446–506)	933 (737–1,157)
	1	293 (266–320)	719 (673–767)	1,164 (954–1,402)
	2	383 (350–417)	1,167 (1,078–1,261)	1,874 (1,598–2,186)
	3	595 (528–665)	1,991 (1,764–2,243)	2,855 (2,429–3,345)
	4	903 (743–1,081)	3,551 (2,979–4,252)	5,337 (4,509–6,338)
	5	3,428 (1,665–7,371)	13,660 (9,612–21,919)	21,775 (17,841–27,517)
	All	365 (347–382)	774 (747–802)	2,258 (2,097–2,430)
**Stroke**	0	259 (247–272)	457 (442–472)	787 (713–864)
	1	351 (337–365)	703 (679–728)	1,189 (1,097–1,286)
	2	458 (441–476)	1,111 (1,065–1,158)	1,721 (1,607–1,841)
	3	707 (675–740)	1,959 (1,838-2,088)	3,100 (2,893–3,320)
	4	1,097 (1,018–1,181)	3,519 (3,224–3,846)	6,044 (5,632–6,490)
	5	3,572 (2,851–4,501)	13,762 (11,583–16,748)	19,003 (17,436–20,825)
	All	427 (419–436)	753 (739-767)	2,305 (2,235–2,376)
**Traumatic Brain Injury**	0	227 (200–256)	388 (355–422)	601 (471–746)
	1	316 (283–351)	694 (634–756)	980 (785–1,203)
	2	417 (375–461)	1,077 (965–1,196)	1,303 (1,060–1,579)
	3	619 (541-701)	1,743 (1,476–2,049)	2,526 (2,068–3,066)
	4	1,039 (838–1,266)	3,041 (2,400–3,870)	4,346 (3,472–5,456)
	5	4,224 (1,984–9,411)	14,425 (9,478–25,812)	16,374 (12,611–22,529)
	All	373 (353–393)	680 (648–713)	1,580 (1,447–1,722)
**None of Above 11 Conditions**	0	170 (163–177)	598 (575–622)	872 (772–978)
	1	286 (277–294)	991 (954–1029)	1376 (1269–1489)
	2	503 (489–518)	1,597 (1,534–1,663)	2,605 (2,455–2,762)
	3	933 (904–962)	2,531 (2,388–2,683)	5,225 (4,973–5,491)
	4	1,885 (1,784–1,990)	4,309 (3,989–4,660)	10,492 (10,044–10,968)
	5	4,097 (3,584–4,699)	10,798 (9,262–12,794)	21,546 (20,543–22,633)
	All	403 (397–409)	1,112 (1,090–1,134)	4,718 (4,610–4,830)

## Discussion

The present study provided strong evidence for the predictive validity of the CHESS scale with regard to survival in a variety of settings and populations. Higher CHESS scores were strong predictors of mortality in home care, nursing home and complex continuing care hospital settings. This trend was evident in the overall population, among persons with neurological conditions as a general category, and also within each of the 11 diagnoses considered here. The only exceptions to these trends were likely the result of reduced cell sizes for certain conditions. For example, the small sample size for Huntington's in HC and CCC may have resulted in insufficient power for analyses of CHESS in those settings.

There are a number of ways in which CHESS may prove useful for clinical, administrative, policy-development, evaluation and research purposes. Clearly, the present results suggest that CHESS scores can provide meaningful clinical insights that would be relevant to care planning and service delivery for persons with neurological conditions. For example, CHESS can identify those who may have reversible instability and who require immediate attention (see, for example, the examination of CHESS in heart failure patients [20]). It can also inform discussions related to prognosis and it may be used as a severity measure to describe the level of instability in the person's health status. The intensity of services offered or the frequency of monitoring or reassessment may be guided, at least in part, based on these scores. Administrative and policy related applications may include the use of CHESS in case mix systems for predicting resource intensity or as a consideration in eligibility systems or targeting criteria for services involving different levels of clinical expertise. An important benefit of the CHESS distribution is that the populations of greatest concern, those with higher CHESS scores, are a relatively small group. Therefore using CHESS for eligibility or targeting criteria produces a manageable number of individuals to focus on for intensive services and advanced care planning. Research and evaluation studies on clinical outcomes related to neurological conditions could reasonably use CHESS as a covariate to adjust for differences in medical complexity or health instability. This would be important for any performance measurement initiatives that would aim to evaluate the quality of care for persons with the conditions considered here.

Most of the associations of neurological conditions with mortality were modest compared to those for age and CHESS. In part this may be because the reference group for each neurological condition (coded as a series of binary variables) likely included other neurological conditions and serious non-neurological conditions (e.g., heart failure, cancer). Also, the notable heterogeneity of sociodemographic and clinical characteristic between neurological diagnoses and across care settings clearly points to the need for information beyond diagnosis alone when considering vulnerable populations across the continuum of care. Indeed, the results presented here suggest that these other covariates that deal with underlying frailty are considerably more important than diagnosis alone when examining trends in mortality in HC, LTC and CCC settings. In addition to CHESS scores, clinical variables that could be considered include gait speed and impairments in activities of daily living, both of which have been shown to predict mortality in older persons [9], [30].

Although there have been some algorithms developed to predict mortality in specific diagnostic groups, the CHESS scale has a number of advantages over more specialized solutions reported elsewhere. First, the CHESS score is embedded within most of the instruments within the interRAI suite of assessments. Hence, any jurisdiction that implements these instruments for their various applications, including care planning, quality monitoring, outcome measurement and resource allocation will by default have CHESS scores available for all persons assessed with these instruments with no additional cost in staff time. The broad national and international use of the interRAI instruments will therefore also permit cross-jurisdictional comparisons of the experiences of person with neurological conditions after adjusting for factors like CHESS scores. The ability to use CHESS as a generic algorithm to predict survival is also a distinct advantage over disease-specific solutions because this allows for direct comparisons across diagnostic groups. Moreover, many disease-specific algorithms include items that are a function of the health system (e.g., diagnostic delay) rather than clinical characteristics of the person making their cross-national utility limited.

The CHESS score should also be considered to be a dynamic clinical measure. Unlike static measures such as the age of onset of symptoms, the items that comprise the CHESS score (e.g., shortness of breath) may be modifiable. Future research should examine the use of CHESS as a time dependent covariate to determine the impact of changes in CHESS score over time.

The cross-sector differences in the associations of various risk factors with mortality may reflect, at least in part, differences in the reference populations against whom persons with neurological conditions are compared. For example, ALS has an adjusted hazard ratio of 1.97 in home care, but less than 1.20 in complex continuing care and nursing homes. In those latter two settings, there is a more uniformly high rate of impairment in all service recipients, whereas there is a greater degree of variation between the least and the most impaired persons in home care. For example, the sample descriptions in Table 1 showed that persons with ALS in Home Care had an almost six times higher percentage of individuals with moderate or worse ADL impairment compared with the non-neurological population. The corresponding ratios in CCC hospitals/units and nursing homes were 1.4 and 1.5, respectively. Hence, diagnosis appears less useful as a predictor of mortality in more intensive care settings, but CHESS appears to perform consistently well irrespective of care setting.

The present study also demonstrates the diversity of persons with neurological conditions. There are marked differences in the mortality experience, demographic and clinical characteristics between diagnostic groups indicating that they should not be considered to represent a collectively homogeneous population. However, it is also clear that there are notable differences in the mortality experience of persons with the same neurological diagnosis *within* and *between* care settings. This diversity within diagnosis points to the need for comprehensive assessment as a basis for care planning and service provision.

The fact that CHESS is not a biological based marker may be a limitation given that it relies on subjective clinical appraisals for at least some scale component rather than more objective physiological measures. Several studies have examined the inter-rater reliability for these measures, and they consistently meet standards for acceptable levels of agreement [23], [25]. However, these instruments do depend on good training, effective communication, and appropriate clinical skills to evaluate the person's health status. It should be regarded as a strength that interRAI assessments can be used to inform clinical practice (i.e., care planning and person level outcome measurement) and management decisions (e.g., quality indicators, case mix classification), because the countervailing incentives for these different applications would tend to balance each other out. On the other hand, if the assessment were used only for a single application (e.g., funding) one might expect that clinical measures like CHESS would become less effective as predictors of health outcomes in these settings. Therefore, it is essential to put in place mechanisms to ensure the quality of data gathered as part of the routine assessment process.

## Conclusions

The CHESS scale provides a useful indicator of mortality risk for persons with neurological conditions in different health service settings across the continuum of care. It differentiates mortality risk within the overall population, those with neurological conditions and specific neurological subgroups. CHESS provides valuable information that clinicians, service providers, policy makers and researchers can use to inform decisions related to the care of persons with neurological conditions.
